# Wogonin suppresses stem cell-like traits of CD133 positive osteosarcoma cell via inhibiting matrix metallopeptidase-9 expression

**DOI:** 10.1186/s12906-017-1788-y

**Published:** 2017-06-12

**Authors:** Do Luong Huynh, Taeho Kwon, Jiao Jiao Zhang, Neelesh Sharma, Meeta Gera, Mrinmoy Ghosh, Nameun Kim, Somi Kim Cho, Dong Sun Lee, Yang Ho Park, Dong Kee Jeong

**Affiliations:** 1 Laboratory of Animal Genetic Engineering and Stem Cell Biology, Department of Animal Biotechnology and Next Generation Convergence Technology, Jeju National University, Jeju, 63243 Republic of Korea; 2 Laboratory of Animal Genetic Engineering and Stem Cell Biology, Subtropical/Tropical Organism Gene Bank, Jeju National University, Jeju, 63243 Republic of Korea; 3 Division of Veterinary Medicine, Faculty of Veterinary Science and Animal Husbandry, Sher-e-Kashmir University of Agriculture Science & Technology of Jammu, R.S. Pura, Jammu, J&K 181102 India; 4 Faculty of Biotechnology, College of Applied Life Sciences, SARI, Jeju National University, Jeju, 63243 Republic of Korea; 5 BRM Institute, Seoul, 135-010 Republic of Korea; 6 Department of Animal Biotechnology, Faculty of Biotechnology, Jeju National University, 102 Jejudaehak-ro, Jeju-si, Jeju-do 63243 Republic of Korea

**Keywords:** Wogonin, *Scutellaria baicalensis*, Osteosarcoma, Cancer stem cells, Mobility

## Abstract

**Background:**

Several efforts have been deployed to cure osteosarcoma, a high-grade malignant bone tumour in children and adolescents. However, some challenges such as drug resistance, relapse, and tumour metastasis remain owing to the existence of cancer stem cells (CSC). There is an urgent need to develop cost-effective and safe therapies.

**Methods:**

Wogonin, an extract from the root of *Scutellaria baicalensis*, has long been considered as a promising natural and safe compound for anti-tumourigenesis, particularly to inhibit tumour invasion and metastasis. Hoechst 33,342 staining, wound healing assay, sphere formation assay, western blotting, and gelatin zymography assays were performed in CD133 positive osteosarcoma cell.

**Results:**

In this study, we examined the effect of Wogonin on the mobility of human osteosarcoma CSC. Wogonin induces apoptosis of human osteosarcoma CSC, inhibits its mobility in vitro via downregulation of MMP-9 expression, and represses its renewal ability.

**Conclusions:**

We demonstrated that Wogonin decreases the renewal capacity of CSC. By inhibiting the formation of and reducing the size of spheres, Wogonin at a concentration of 40–80 μM effectively minimizes potential risk from CSC. Taken together, we have demonstrated a new approach for developing a potential therapy for osteosarcoma.

## Background

Osteosarcoma is a high-grade malignant bone tumour in children and adolescents that possesses the potential for early metastasis [1]. Genetic alterations or epigenetics interrupt the differentiation of mesenchymal stem cells into osteoblasts [2]. As a result, these malignant osteoblasts produce immature bone or osteoid tissue at the active sites of bone growth such as the femur, tibia, and humerus [1]. Several efforts have been deployed to cure osteosarcoma, which combine surgery and various chemotherapies or radiation [1–3]. However, these strategies are not completely effective and meet with difficulties such as drug resistance, relapse, and, particularly, tumour metastasis [4, 5].

The primary cause of death in patients with osteosarcoma is pulmonary metastasis [4], as also reported by Geller et al. [5], Moreover, the capability of cancer stem cells (CSC) to cause tumour relapse is of great concern. These risks are latent and lead to poor diagnoses. Thus, the prevention of metastasis in osteosarcoma CSC is imperative.

Matrix metalloproteinases (MMP) are involved in the invasion and metastasis of cancer in general, particularly in osteosarcoma [6, 7]. With the ability to degrade the basal lamina of the extracellular matrix (ECM), MMP play an important role in facilitating tumour invasion and metastasis. In addition, MMP promote the formation of new blood vessels at the tumour sites, a process known as angiogenesis, and participate in epithelial–mesenchymal transition (EMT) [8]. Thus, it is necessary to counteract the activity of MMP.

Wogonin, an active compound found in *Scutellaria baicalensis*, is a well-known anticancer drug used for various types of cancers, including hepatic carcinoma, pulmonary carcinoma, and glioblastoma [9]. Some evidences have illustrated that Wogonin effectively inhibits the invasion and migration of human gallbladder carcinoma [10], breast cancer [11], oral cancer [12], and melanoma [13], but there is no evidence of its effect on osteosarcoma. In this study, we aimed to evaluate the inhibitory effect of Wogonin on the invasion of CD133^+^ Cal72 human osteosarcoma stem cell, a critical step in metastasis of the tumour exhibited by the downregulation of MMP-9, and to elucidate the effects of Wogonin on the sphere formation ability of CSC to design a novel, natural, and safer anti-mobility and anti-invasion agent.

## Methods

### Reagents

Wogonin (purity >98%) was purchased from Sigma-Aldrich (St. Louis, MO), dissolved in dimethyl sulphoxide (DMSO; Sigma-Aldrich, St. Louis, MO), and stored at −20 °C. Polyclonal antibodies against Bcl-2, Bax, p53, poly ADP ribose polymerase (PARP), caspase-3, and MMP-9 antibodies, and horseradish peroxidase (HRP)-conjugated anti-rabbit or anti-mouse immunoglobulin G (IgG) were purchased from Santa Cruz Biotechnology (California, USA).

### Cell culture

CD133^+^ CAL72 cells were cultured [14] in Dulbecco’s modified Eagle’s medium (DMEM)/F12 (Gibco, NY, USA) supplemented with 10% foetal bovine serum (FBS; Gibco), 20 ng/mL epidermal growth factor (EGF; Sigma-Aldrich), and 20 ng/mL *basic fibroblast growth factor* (bFGF; KOMA biotech, Seoul) and maintained in a humidified atmosphere of 5% CO_2_ in an incubator at 37 °C, by passaging two times every week.

### Hoechst 33342 staining

CD133^+^ CAL72 cells were cultured in 12-well plates and exposed to varying concentrations of Wogonin (0, 10, 20, 40, and 80 μM) for 24 h and then incubated with Hoechst 33342. Fluorescence microscopy was used to observe the cell shape captured from five random visual fields.

### Wound healing assay

CD133^+^ CAL72 cells were used for wound healing assay as previously described [15]. In brief, cells at log phase were seeded into a 12-well plate at 98%–100% confluence. After scraping using a sterile yellow tip, unattached cells were washed twice with the medium and replaced with 0.5 mL of fresh medium with only the complete medium (negative control) or different concentrations of Wogonin for 24 h. The cell-free zone was captured by an inverted microscope, and five randomly chosen fields were analysed for each well. The assay was independently repeated in triplicates.

### In vitro cell migration and invasion assays

Cell migration assay was performed using 8-μm pore size trans-well inserts (Costar, Cambridge, MA, USA). In brief, the lower chamber was filled with 0.6 mL of DMEM (Gibco, NY, USA) supplemented with 20% FBS (Gibco). Cells were starved overnight, the medium was supplemented with 0.5% FBS, cells were harvested, and washed twice with serum-free DMEM. Cells with the concentration of 1 × 10^5^ in 0.1 mL of DMEM with 0.5% FBS were added to the upper chamber. After incubation for 24 h, cells on the upper surface of the membrane were detached using cotton swabs. The migrant cells attached to the lower surface were fixed in methanol at room temperature for 30 min and stained for 20 min with a solution containing 0.5% crystal violet. The number of cells that migrated on the lower surface of the membrane was counted under a microscope in five random fields at 100×. For cell invasion assay, all procedures were performed as in the migration assay, except that a Matrigel matrix growth factor (with reduced basement) at a concentration of 3.5 mg/mL (BD Biosciences) was coated on the upper chamber according to the manufacturer’s protocol, followed by incubation at 37 °C for 2 h before culturing the cells on the upper chamber. To determine the absorbance by crystal violet, the stained cells were flooded with 100% methanol for 10 min followed by measuring the OD at 573 nm.

### Colony assay

Cells were seeded at 1 × 10^3^ cells per well in a six-well plate and cultured in DMEM (Gibco) containing 10% FBS (Gibco) and 1% antimycotic (Thermo Fisher Scientific). After 2 h for attachment, single cells were treated with varying concentrations of Wogonin (0, 10, 20, 40, and 80 μM) and incubated at 37 °C and 5% CO_2_ until the wells in the negative control group developed visual colonies. The colonies were then fixed with methanol–acetic acid (3:1) for 20 min and stained with 0.5% crystal violet for 15 min at room temperature.

### Sphere formation assay

CD133^+^ CAL72 cells were trypsinized, followed by sieving with a 40-μm cell strainer, and seeding into a 96-well ultralow attachment plate at 1 × 10^3^ cells per well in DMEM/F12 medium supplemented with 0.4% bovine serum albumin (BSA; Bioprince, Gangwon-do, Korea), 20 ng/mL EGF (Sigma-Aldrich), 20 ng/mL bFGF (KOMA biotech). Wogonin was then supplemented at appropriate concentrations (0, 10, 20, 40, and 80 μM), and the plate was incubated for 9 days.

### Western blotting

A total of 1 × 10^6^ cells were collected and lysed in radioimmunoprecipitation assay (RIPA) buffer (Sc-24,948, Santa cruz Biotech), according the manufacturer’s protocol. Total proteins were subjected to 12% sodium dodecyl sulphate–polyacrylamide gel electrophoresis (SDS–PAGE), transferred electrophoretically (Bio-Rad, PA, USA) onto a polyvinylidene fluoride (PVDF, Bio-Rad, PA, USA) membrane, and blocked with 5% non-fat milk powder (*w*/*v*) in 1× phosphate buffer saline with Tween 20 (PBST) for 1 h at room temperature. The membranes were incubated with primary antibodies or with anti-β-actin mouse polyclonal antibody as an internal control overnight at 4 °C. HRP-conjugated anti-rabbit or anti-mouse secondary antibodies were incubated at room temperature for 2 h. The bands were captured by ImageQuant™ LAS 4000 mini Fujifilm.

### Gelatin zymography

CD133^+^ CAL72 cells were treated with 10, 20, 40, and 80 μM Wogonin and with 0.1% DMSO (Sigma-Aldrich) as the negative control in the same amount of medium supplemented with 0.5% FBS (Gibco) for 24 h. Then, 10 μL of the supernatant of the medium from each group was collected and mixed at a ratio of 3:1 with a non-reducing loading buffer without heating. After settling for 5 min at room temperature, the protein samples were subjected to 10% SDS–PAGE–0.1% gelatin (Bio-rad), followed by washing twice for 20 min in 2.5% Triton X-100 solution to remove SDS. Subsequently, the washed gel was incubated in a developing buffer (50 mM Tris-HCl pH 7.6, 5 mM CaCl_2_, 0.2 M NaCl, 0.02% *w*/*v* NaN_3_) at 37 °C for 24 h. Then, the gel was stained with 0.1% Coomassie Brilliant Blue G250 (Biorad) for 1 h, followed by destaining for 2 h in 10% acetic acid and 10% methanol. The stained gel was measured by ImageJ software for relative quantification.

### Statistical analysis

Data are expressed as mean ± standard error of measurement (SEM). Experimental differences were examined using ANOVA and Student’s t-tests, as appropriate. *P* values of <0.05 were considered to indicate statistical significance.

## Results

### Wogonin sensitizes CD133^+^ CAL72 cells for apoptosis

**Fig. 1:**
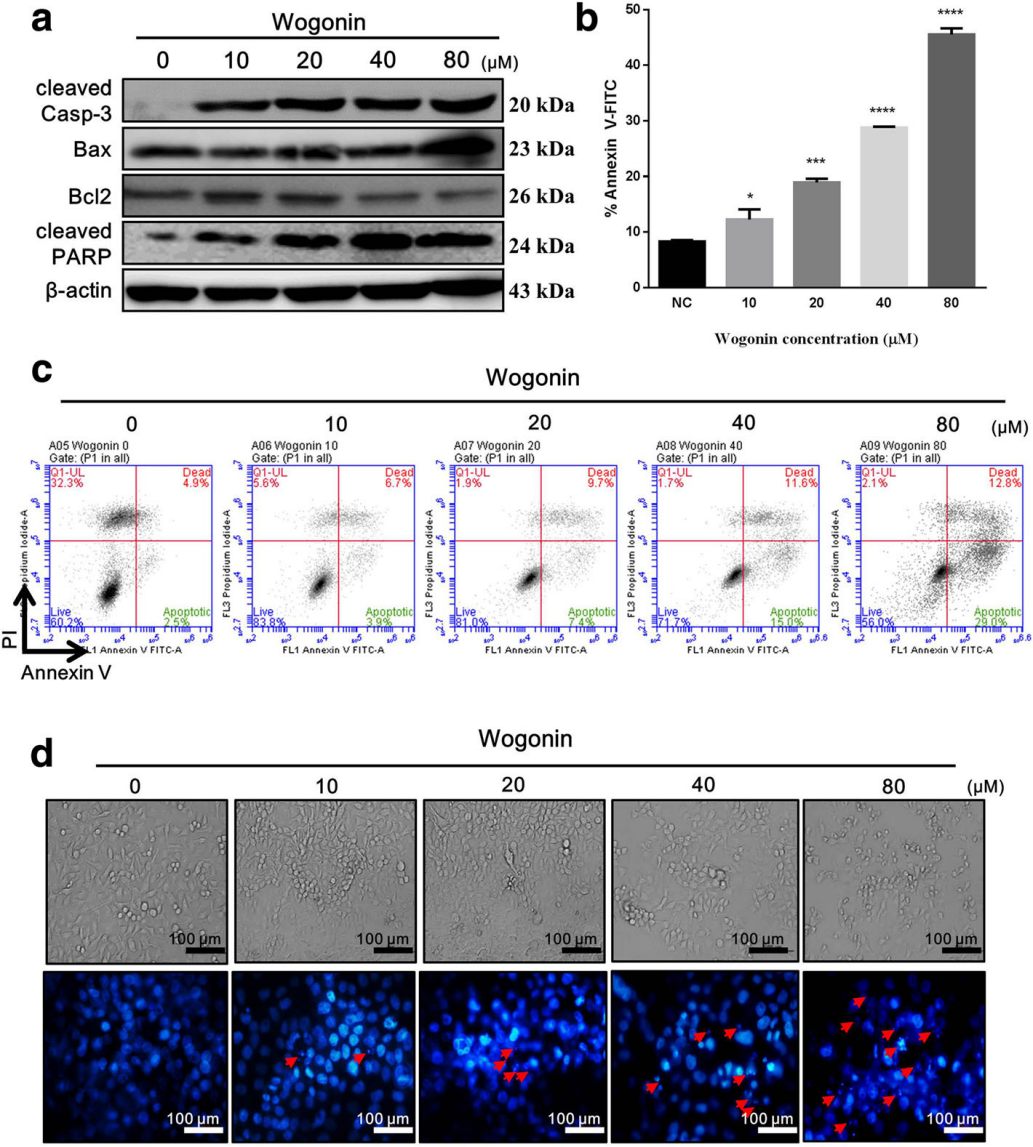
Wogonin induces apoptosis in CD133^+^ CAL72 CSC. **a** Western blotting was performed on 24 h-treated extract of CSC in the presence of varying concentrations of Wogonin (0, 10, 20, 40, 80 μM). **b** Percentage of annexin-V-positive cells after Wogonin treatment. **c** Percentage of apoptotic cells as analysed by flowcytometry. **d** The morphology of CSC was altered at 24 h post treatment; the changes in nuclear morphology were observed by Hoechst 33,342 staining after treating with the indicated Wogonin concentration (0, 10, 20, 40, 80 μM). All experiments were performed in duplicates. Data are represented as mean ± SEM (*n* = 3). ^*∗*^ *P <* 0.05, ^*∗∗*^ *P <* 0.01, ^****^ *P <* 0.0001

Previous studies have shown that Wogonin exhibits antiproliferative activity against cancer cells via intrinsic apoptosis [9]. Further analysis indicated that Wogonin triggers the activation of apoptotic markers such as caspase-3 and Bax and inhibits Bcl2 and PARP function [9]. In this study, we hypothesized that human osteosarcoma CSC, which were isolated by Mongre et al. [14], are considered as the main determinant in osteosarcoma relapse [16] and are probably suppressed by Wogonin. Therefore, to investigate whether Wogonin induces apoptosis of CD133^+^ CAL72 CSC, we exposed the cells to various concentrations of Wogonin for 24 h. As shown in Fig. 1, apoptosis probably occurred during the treatment because activation of caspase-3 and Bax, suppression of Bcl2, and cleavage of PARP were observed (Fig. 1a). Moreover, the level of annexin-V positive cells was determined by fluorescence-activated cell sorting (FACS) analysis after 24 h of treatment. As demonstrated in Fig. 1b and c, a dose-dependent increase was observed in the levels of annexin V/propidium iodide (PI)-positive cells along with the level of apoptotic cells. Furthermore, the morphology of cells exposed to varying concentrations of Wogonin was altered, and the cells detached from the culture plate surface (Fig. 1d). In the presence of Hoechst 33,342, changes in the nucleus were observed upon exposure to Wogonin (Fig. 1d). Taken together, these data suggest that Wogonin induces apoptosis of CD133^+^ CAL72 cells via the mitochondrial pathway related to Bax/Bcl2 axis.

### Wogonin inhibits the migration of CD133^+^ CAL72 CSC

**Fig. 2:**
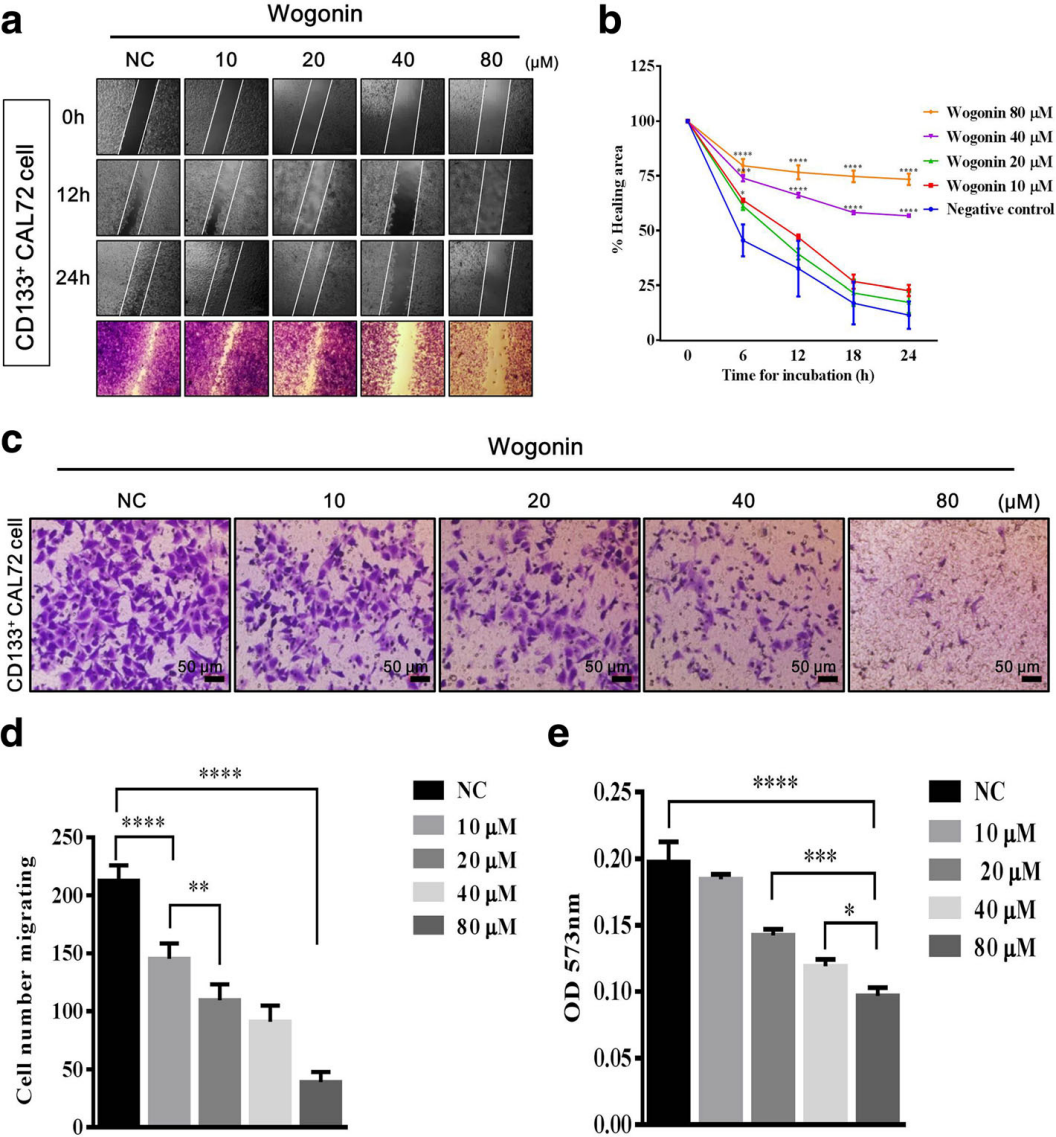
Effect of Wogonin on the migration ability of CD133^+^ CAL72 CSC. **a** Wound healing assay showed that the rate of healing decreased after Wogonin treatment (0, 10, 20, 40, and 80 μM). **b** The percentage of healing area was constrained by Wogonin treatment (0, 10, 20, 40, and 80 μM), as measured by ImageJ software at 24 h post treatment. **c** Migration of CD133^+^ CAL72 cells was inhibited by Wogonin treatment (0, 10, 20, 40, and 80 μM), as visualized by staining with 0.5% crystal violet. **d** The number of migrating cells was evaluated in five random fields in each trans-well insert exposed to varying concentrations of Wogonin (0, 10, 20, 40, and 80 μM). **e** OD_573nm_ measurement after destaining with methanol and acetic acid. All experiments were performed in triplicates. Data are represented as mean ± SEM (*n* = 3). ^*∗*^ *P <* 0.05, ^*∗∗*^ *P <* 0.01, ^***^ *P <* 0.001, ^****^ *P* < 0.0001

Extensive evidence has suggested that CSC tend to be mobile and metastasize to form a new tumour residing at ‘oasis land’ [17]. Therefore, prevention of CSC mobility is indispensable. To investigate whether Wogonin suppresses the migration property of human osteosarcoma CSC, CD133^+^ CAL72 cells were treated with varying concentrations of Wogonin (0, 10, 20, 40, and 80 μM) during the 24-h wound healing assay. As shown in Fig. 2a and b, it was clearly observed that the gap closures were intercepted significantly in the presence of 40–80 μM Wogonin. Next, we performed a migration assay to examine CSC mobility after exposure to Wogonin. Fig. 2c shows that the number of cells pretreated with Wogonin (0, 10, 20, 40, and 80 μM) migrating through the cell culture insert membrane were significantly diminished in a dose-dependent manner. Microscopic observation after crystal violet staining indicated that Wogonin effectively inhibited the mobility of human osteosarcoma CSC (Fig. 2d). Furthermore, OD_573nm_ measurement demonstrated a decrease in cells stained with crystal violet (Fig. 2e). These evidences suggest that Wogonin enfeebles migration of human osteosarcoma CSC.

### Wogonin participates in anti-invasion of CD133^+^ CAL72 CSC

**Fig. 3:**
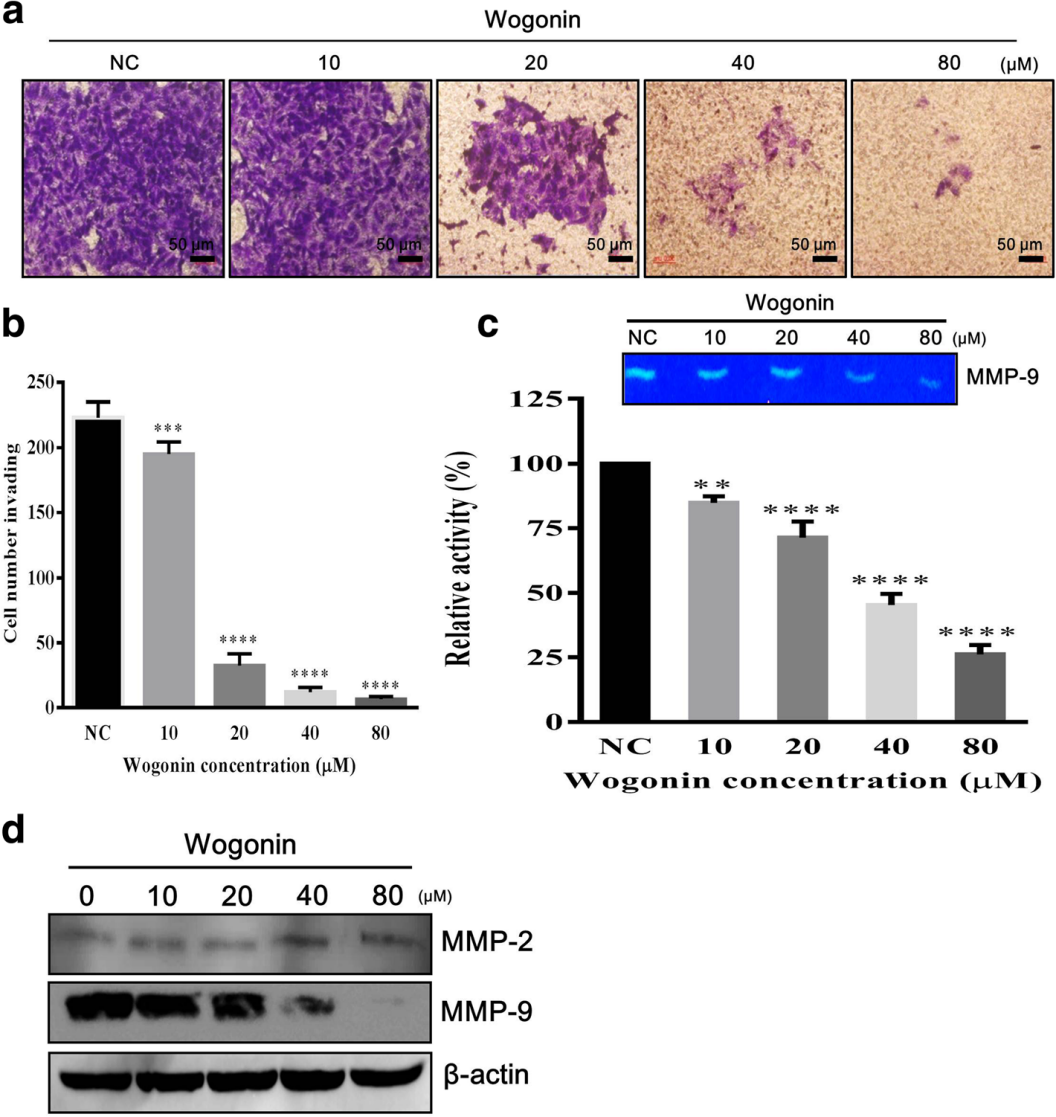
Wogonin inhibits the invasion potential of CD133^+^ CAL72 CSC. **a** Cells were treated with varying concentrations of Wogonin (0, 10, 20, 40, and 80 μM) 24 h prior to the invasion assay, followed by staining with 0.5% crystal violet. **b** Invading cells were calculated by counting cells in five random fields under the microscope. **c** Supernatant of the medium containing cultured Wogonin-treated human osteosarcoma CSC was subjected to gelatin zymography assay. **d** Western blot assay of total extract proteins derived from 24-h treated CSC exposed to varying concentrations of Wogonin (0, 10, 20, 40, and 80 μM). Data are represented as mean ± SEM (*n* = 3). ^*∗*^ *P <* 0.05, ^*∗∗*^ *P* < 0.01, ^***^ *P* < 0.001, ^****^ *P* < 0.0001

One of the important characteristic events of cancer progression is invasion of the surrounding tissues by the tumour. CSC play a key role in tumour metastasis and maintain the tumour microenvironment for efficient tumour growth and EMT [18, 19]. Therefore, targeting CSC invasion is crucial for inhibiting tumour expansion. To clarify the effect of Wogonin on the invasion property of osteosarcoma CSC, varying concentrations of Wogonin were supplemented into the mixture of cells and medium on Matrigel. As shown in Fig. 3a and b, Wogonin at the concentration of 20–80 μM significantly inhibited the invasion by CSC.

Furthermore, we evaluated the effect of Wogonin on MMP proteins in CSC. MMP proteins determine and regulate the tumour environment [20] and represent the capability of tumour invasion and metastasis [8]. In this study, both cell extracts and conditioned medium were subjected to western blotting and gelatin zymography assays, respectively. Because gelatin is a substrate of gelatinases (MMP-2 and MMP-9), it probably determines the capacity of MMP-9 activity, as demonstrated by gelatin zymography. In 10% SDS–PAGE gel premixed with gelatin, the level of MMP-9 activity significantly decreased when Wogonin was supplemented in the range of 40–80 μM (Fig. 3c). The level of bands was then assessed by the ImageJ software, and it was observed that the relative activity decreased by 25% on exposure to 80 μM Wogonin compared to the negative control (NC). Moreover, western blot data indicated that level of MMP-9 decreased efficiently, interpreting the retrogression of CSC mobility (Fig. 3d). Taken together, these data suggest that Wogonin efficiently constrains the invasion of CD133^+^ CAL72 CSC.

### Wogonin suppresses the self-renewal capacity of CD133^+^ CAL72 CSC

**Fig. 4:**
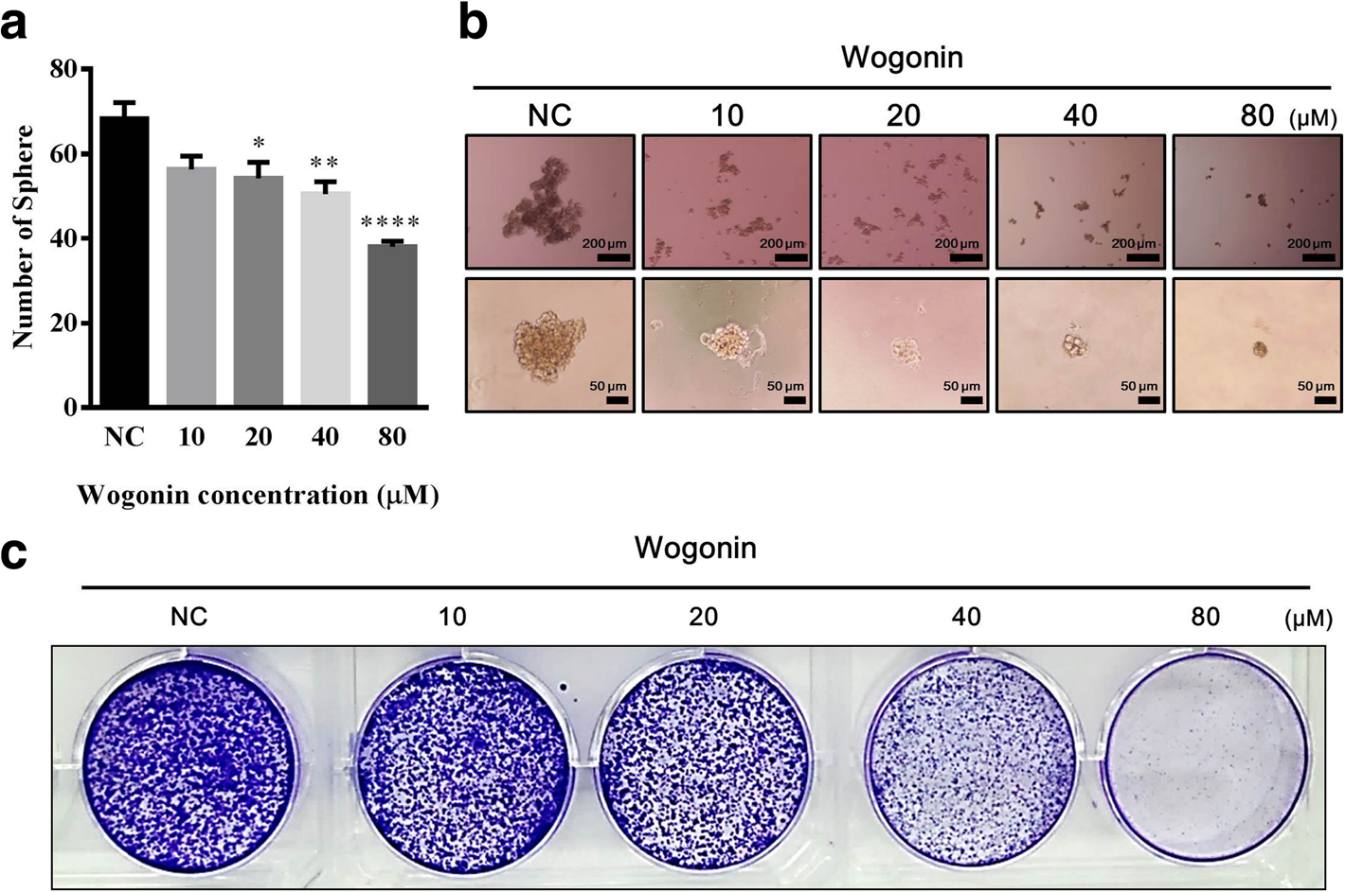
Effects of Wogonin on the renewal capacity of CD133^+^ CAL72 CSC. **a** The effect of Wogonin on inhibiting the number of spheres. **b** Single sphere formation of CD133^+^ CAL72 cells after 9 days in suspension culture containing Wogonin (0, 10, 20, 40, and 80 μM). **c** Wogonin reduced the clonogenicity of CD133^+^ CAL72 cells; each well was seeded with 1 × 10^3^ cells and incubated at 37 °C with 5% CO_2_ after 24 h of Wogonin treatment. Then, the colonies were visualized by staining with 0.5% crystal violet. Data are represented as mean ± SEM (*n* = 3). ^*∗*^ *P* < 0.05, ^*∗∗*^ *P* < 0.01, ^****^ *P* < 0.0001

With their ability to repopulate the tumour from a single cell, CSC pose a latent risk in cancer treatment because of their resistance to anticancer therapies [21]. Therefore, inhibition of the self-renewal capacity is prerequisite for CSC treatment. In this study, we examined whether Wogonin inhibits the self-renewal capacity of osteosarcoma CSC. As shown in Fig. 4a, the number of spheres formed in the suspension culture medium decreased significantly, particularly in the presence of 40–80 μM Wogonin. In terms of the sphere size, Wogonin significantly diminished the growth of the sphere (Fig. 4b). In the colony assay, single cells forming colonies were inhibited at Wogonin concentration of 40–80 μM (Fig. 4c). These results suggest that Wogonin interferes with the basic characteristics of CD133^+^ CAL72 CSC, facilitating the elimination of CSC in tumour microenvironment.

## Discussion

During tumourigenesis, the maintenance of oxygen and energy supply for the rapid growth of cells is vital. Among the typical characteristics of cancer, which have been described by Hanahan et al. [19], MMP proteins present as crucial factors regulating different stages of the tumour [8]. MMP play a role in tumour invasion as more space is required, leading to the degradation of ECM and facilitating tumour invasion and metastasis. Moreover, MMP also help in releasing some growth factor precursors such as insulin-like growth factor (IGF) and EGF, which enhance cell proliferation. As the tumour volume increases, the need for blood supply is also considerable. Angiogenesis promoted by MMP allows formation of new blood vessels that surround the tumour mass, by which the tumour tissue is remodelled. Furthermore, MMP also show effects in either immune surveillance or EMT process, and both these processes are pivotal in tumour development.

Our study demonstrated that Wogonin, a compound extracted from *S. baicalensis*, targets the invasion of osteosarcoma CSC through MMP-9 suppression. In fact, Wogonin possesses the ability to avert the mobility of CSC. These effects are observed because of decreased MMP-9 expression during Wogonin exposure. The results of this study are consistent with previous studies reported on human gallbladder carcinoma, breast carcinoma, oral cancer, melanoma, and inflammation-associated lung carcinoma, which indicate that Wogonin is able to reduce the migration/invasion of cancer cells upon treatment [22].

Some cancer cell lines express both MMP-2 and MMP-9, whereas some cells express only MMP-2 [6]. In this study, the level of MMP-2 could be detected by western blotting, but Wogonin did not affect MMP-2 expression. However, MMP-9 likely plays a role in the interaction of CSC and tumour niches, as opposed to MMP-2. In addition, several studies have suggested that MMP-9 is highly involved in the maintenance of CSC properties inside the tumour microenvironment, and changes in MMP-9 expression may affect the invasion property of CSC [23–26]. Furthermore, previous reports indicated that MMP-9 triggers angiogenesis/ invasion while MMP-2 plays vital role in tumor growth [27, 28]. These evidences indicated different roles and different demands of MMP-2 and MMP-9 expression among tumor growth stages. It is probably that the shift of expression between MMP-2 and MMP-9 state for the transition of primary tumor and invasive tumor. Bjørnland et al. indicated that MMP-9 represents in almost all biopsies including primary tumors, metastases tumors [6]. Li et al. suggested that MMP-9 is biomarker of survival in patients with osteosarcoma [29]. Taken together, these clinical evidences state that MMP-9 is essential for tumor metastasis which makes osteosarcoma become more risky instead of its usual localization. Therefore, in attempt to inhibit the invasion, angiogenesis, metastasis of osteosarcoma, Wogonin has met to anti-cancer expansion via down-regulation of MMP-9. Regarding the renewal capacity, CSC are of great concern in antitumor therapies. CSC help to sustain tumour growth during therapies, leading to a high risk of relapse [22]. However, it is difficult to administer drugs targeted to the bone as the drug cannot approach the tumour niche and loses its pharmacokinetic activity [30]. Therefore, it is necessary to assess the effect of direct Wogonin treatment at osteosarcoma sites to block CSC metastasis at an early stage before unwanted CSC invasion and metastasis. Furthermore, Wogonin exhibits the abilities to prevent osteosarcoma CSC metastasis in the body and/or to preclude the circulating osteosarcoma CSC in the blood stream. The usage of Wogonin as a monotherapy or as a combination therapy with other safe compounds is an ideal option that needs to be examined further.

## Conclusions

In conclusion, identification and extermination of CSC is an essential approach for any therapeutic treatment. We demonstrated that Wogonin decreases the renewal capacity of CSC by inhibiting the formation of and reducing the size of spheres. Furthermore, Wogonin at concentration of 40–80 μM can effectively minimize the potential risk due to CSC. Therefore, Wogonin can be potentially used an anti-cancer drug and may help in decreasing the metastasis of osteosarcoma.

## Abbreviations

CSC: Cancer stem cell
ECM: Extracellular Matrix
EMT: Epithelial–mesenchymal transition
MMP-9: Matrix Metalloproteinases-9
